# Building readiness in community-based organisations to enable the implementation of public health interventions for adults and older adults: a scoping review

**DOI:** 10.1186/s12889-025-25745-y

**Published:** 2025-11-24

**Authors:** Leanne Hassett, Anne M. Moseley, Lindsay Nettlefold, Louise M. N. Pearce, Thea Franke, Heather M. Macdonald, Anne Tiedemann, Heather A. McKay

**Affiliations:** 1 Faculty of Medicine and Health, The University of Sydney, Sydney, Australia; 2 Faculty of Medicine, The University of British Columbia, Vancouver, Canada

**Keywords:** Organisational readiness, Public health, Community, Measurement, Implementation strategies

## Abstract

**Background:**

A key challenge to implementing and scaling up evidence-based interventions (EBIs) into practice is organisational readiness; described as an organisation’s motivation, general capacities, and capabilities specific to the EBI. Building organisational readiness has been investigated in some health disciplines (e.g., mental health). However, the importance of building organisational readiness to effectively implement public health EBIs for adults and older adults in the community setting remains largely unexplored. Our aim was to examine how readiness was defined and measured, what strategies were used to build readiness, and the relationship between readiness-building strategies and implementation, service-level, and person-level outcomes.

**Methods:**

In this scoping review, we searched seven databases and conducted forward and backward citation tracking. From a pool of eight reviewers, combinations of two reviewers independently screened references for eligibility. A single reviewer extracted data, and a second reviewer checked data. Results for each implementation, service-level and person-level outcome in each study were extracted and categorised as favourable, nonsignificant, or unfavourable.

**Results:**

Twelve studies were included, which implemented a mix of different public health EBIs to almost 40,000 participants (*n* = 37,883; 54% women) across varied community settings. Only four studies defined readiness; all used different definitions. Five studies used five different instruments to assess readiness, all with poor psychometric properties. All studies used multiple strategies to build readiness (range 4–20 strategies per study), with all using strategies to assess, plan and monitor implementation of the EBI (i.e., ‘evaluative and iterative strategies’) and strategies to support collaboration between organisations delivering the EBI (i.e., ‘develop interest-holder interrelationships’). Three-quarters of the strategies focused on building the organisation’s capability to deliver the specific EBI (e.g., assessing readiness, conducting educational meetings) and were delivered by external support teams. Exploring the relationship between readiness-building strategies and study outcomes indicated more favourable than unfavourable outcomes, particularly for implementation and service-level outcomes (38/48; 79% favourable).

**Conclusions:**

Within this limited sample, the use of readiness-building strategies improved the implementation of public health EBIs in community organisations. However, consistency of definitions and terminology and more sophisticated testing of readiness-building strategies will help confirm how best to do this.

**Trial registration:**

Open Science Framework, May 5, 2024.

**Supplementary Information:**

The online version contains supplementary material available at 10.1186/s12889-025-25745-y.

## Background

Community-based organisations are often tasked with implementing public health evidence-based interventions (EBIs). However, effectively implementing EBIs remains a persistent challenge, as barriers to doing so are complex and surface at many levels (e.g., funding and resources [1], staff attitudes [2]). As a result, most EBIs are never implemented into routine practice [3, 4]; a staggering 75% of attempts to implement them do not succeed [5, 6]. 

Foremost among implementation challenges is an organisation’s readiness to implement an EBI. Organisational readiness (‘readiness’) is key to implementation success [7–9]. We define readiness as “tangible and immediate indicators of organizational commitment to its decision to implement an intervention” [10]. The heuristic (R = MC^2^), used to describe readiness, was generated from compiling determinants of successful implementation across disciplines [11]. The heuristic comprises three primary components—organisation’s motivation (M), general capacities (C), and innovation-specific capacities (C) to implement an EBI. This dynamic, continuous readiness construct is linked to 18 subcomponents that are measurable and can be altered using capacity-building approaches (e.g., tools, training, technical support) [11]. The R = MC^2^ heuristic has gained popularity [12, 13], while other constructs have also been used to define an organisation’s readiness to implement (e.g., change acceptance) [8]. 

Implementation support systems (also called central support units [14]) can be internal or external to an organisation, and are often tasked with evaluating if, and to what extent, organisations are motivated and have the capacity to adopt and implement an EBI. Once gaps in readiness are identified, support units can select, tailor, and apply implementation strategies to fill those gaps [11]. Despite this key role, too few studies delve into the essential role of support units in successful implementation of an EBI.

Implementation strategies (‘strategies’) are methods or techniques used to enhance the adoption, implementation, and sustainability of an EBI [15]. Among the Expert Recommendations for Implementing Change (ERIC) taxonomy of 73 discrete strategies [15], 48 were considered relevant to promote readiness [2]. In mental health services, strategies that build readiness (i.e., engagement of leadership, access to knowledge and information) were commonly included as part of implementation processes [16]. These strategies were used in child, adolescent and adult mental health teams in different settings (e.g., schools, child welfare, psychiatric clinics) and clinical interventions (e.g., parent-child interaction therapy, guidelines for depression and suicidal behaviours). While data could not be pooled, positive results were reported for implementation or clinical outcomes in most trials. In contrast, in public health we could find no reviews of how strategies were utilised at different timepoints and whether or how they build readiness.

Many instruments (approximately 130) in health policy making, public health, health services, social services, and non-health settings have been used to quantify an organisation’s readiness to implement an EBI [1, 8, 17–20]. These are murky waters in implementation science, as there is no standard approach to naming instruments (or versions of instruments) [21]. The lack of standardisation creates problems, as surveys with the same name might include different items based on their genesis from different implementation science frameworks, settings, or level of analysis. Selecting the most appropriate instrument is also confounded by the fact that few readiness instruments undergo rigorous psychometric testing [1, 8, 17, 20]. 

We sought to fill gaps in the literature by conducting a review to ascertain the role that readiness plays in implementing public health EBIs for adults and older adults. This review aligns with a recently funded Canada-Australia initiative (Canadian Institutes of Health Research and Australian National Health and Medical Research Council) that proposed to adapt, implement, and scale-up an effective health-promoting program (Choose to Move; https://www.choosetomove.ca/) [14, 22–26].

Our overarching aim is to determine whether and how readiness is used to guide implementation of public health EBIs in community settings for adults and older adults. In this context we will address the following three research questions:

- Q1. How is readiness defined and measured?
- Q2. What strategies are used to build readiness?
- Q3. What is the relationship between strategies and outcomes? a. For implementation and service-level outcomes? b. For person-level outcomes?

## Methods

### Design

We conducted a scoping review because we wanted to investigate how building readiness is being used to guide implementation of public health EBIs, including identifying the prevalence of strategy use and knowledge gaps. Our review was guided by the Preferred Reporting Items for Systematic reviews and Meta-Analyses extension for Scoping Reviews Statement (PRISMA-ScR) [27] (see Supplementary Material 1) and the Joanna Briggs Institute Manual for Evidence Synthesis [28]. We registered the protocol a priori on the Open Science Framework [29]. Data are available in a public, open access repository (Sydney eScholarship Repository, https://hdl.handle.net/2123/34540, Creative Commons Attribution V.4.0 Licence) [30]. 

### Searches

We searched the following databases on 4 June 2024: Medline (via Ovid); Cochrane Database of Systematic Reviews, Cochrane Central Register of Controlled Trials (CENTRAL), and Cochrane Clinical Answers (via cochranelibrary.com); CINAHL (via EBSCO); PsycINFO (via Ovid); and Embase (via Ovid). The search strategy was developed for Medline and adapted for use within the other databases (see Appendix 1 in the supplementary file [30]). Search terms comprised combinations of the following concepts: organisational readiness for implementation AND public health interventions in community-based settings AND adults and older adults. These searches were developed in close collaboration with experts in the field, by reviewing search strategies used in existing reviews, and by consulting an Academic Liaison Librarian at The University of Sydney. We conducted forward citation tracking of all included studies to identify additional articles and studies published by the authors. We conducted backward citation tracking of relevant systematic or scoping reviews and all included studies to identify additional articles and studies.

### Study inclusion and exclusion criteria

The inclusion and exclusion criteria are listed in Table 1.

**Table 1:** List of inclusion and exclusion criteria

Inclusion criteria 1. Participants predominantly adult and older adult (mean age > 18 years). 2. Public health interventions that provided at a person level. 3. Intervention delivered in a community setting. 4. Assesses readiness for implementation. 5. Describes one or more strategies to build readiness for implementation. 6. Any experimental research design. 7. Published as a full article in a peer-reviewed journal. 8. Any language. 9. Any date of publication. Exclusion criteria 1. Participants predominantly children or adolescents (< 18 years). 2. Public health interventions that involve pharmacology, screening, changing the environment, policy, or mass media education. 3. Health services that deliver health care to individual people in hospital (inpatient) or ambulatory (outpatient) settings. 4. Interventions delivered in the workplace. 5. Systematic reviews and scoping reviews.	

#### Participants

This scoping review included experimental studies in which public health EBIs targeted adults or older adults. We defined adult as 18 years of age or older and older adult as 65 years of age or older.

#### Eligible interventions

Our review focused on public health EBIs conducted in community settings at a person level that involve health promotion or chronic disease management programs that target lifestyle (e.g., Stepping On falls prevention program [31], Choose to Move health promotion program [14, 22–24]). We excluded interventions that were not targeted at the person-level (e.g., changing the environment, policy, mass media education) or were not health promotion or chronic disease management programs targeting lifestyle (e.g., pharmacology, screening). We define public health as “the science and art of preventing disease, prolonging life and promoting human health through organized efforts and informed choices of society, organizations, public and private, communities and individuals” [32]. We define health promotion as “the process of enabling people to increase control over and to improve their health” [33]. Chronic disease prevention programs are “specific efforts aimed at reducing the development and severity of chronic diseases and other morbidities” [34]. We define community settings as locations outside a hospital inpatient or acute care setting; these include home and public or private community centres.

#### Implementation strategy

To be included, studies needed to (i) assess readiness, and (ii) describe one or more strategies used to build readiness. Assessment could involve using a readiness-specific instrument [1, 8, 17–20], a general implementation instrument that included a readiness component (e.g., Consolidated Framework for Implementation Research (CFIR)), or qualitative data (e.g., interviews, focus groups) that were coded to a readiness framework (e.g., R = MC^2^) [11]. Strategies to build readiness for implementation could occur at any time during the implementation process [2]. 

#### Evidence sources

We included full articles published in peer-reviewed journals. Studies adopting any experimental research design (i.e., randomised and non-randomised controlled trials, one-group pre-post studies, single case studies, cross-sectional surveys) were included. There were no restrictions on language or year of publication.

### Potential effect modifiers and reasons for heterogeneity

Not applicable.

### Study quality assessment

Quality assessment was not undertaken as this was a scoping review.

### Data extraction strategy

We screened references using Covidence [35]. Combinations of two reviewers from a pool of eight reviewers independently applied a two-step process to identify relevant studies. In step one, reviewers screened titles and abstracts to identify references that may meet inclusion criteria. In step two, reviewers screened the full text of references selected in step one to ensure they met inclusion criteria. Disagreements were resolved by consensus or by a third reviewer. We pilot tested the study selection process using a random sample of 50 references. We screened references as per inclusion and exclusion criteria, guided by a document that contained definitions and examples (see Appendix 2 in the supplementary file [30]). Independent reviewers met to discuss inclusion or exclusion discrepancies. While we planned to amend the inclusion and exclusion criteria after the pilot, this was not needed.

One reviewer piloted and updated the extraction instrument. One reviewer extracted key information from each study using information reported in the full text article(s) for each study; we did not contact authors for clarification or additional data. A second reviewer checked the extracted data in a random 10% sample of studies. As accuracy exceeded 90%, we reported data generated by a single reviewer. While not pre-specified in our protocol, a second reviewer checked the extraction and coding of all outcomes and strategies for all studies.

To describe the study, the public health EBI and the recipients of the EBI we extracted the following data: citation (first author, year of publication, title, journal); research design (coded as randomised controlled trial, non-randomised controlled trial, one-group pre-post study, single case study, cross-sectional study); publication language; country of implementation; details about the public health EBI, setting, providers, and recipients. We extracted free text descriptions of the intervention and setting and classified interventions as: physical activity; behavioural nutrition; mental health; smoking cessation; multiple (specify); or other (specify). We recorded the number of participating sites and classified the settings as: third sector (i.e., formally structured, independent from government, non-profit, self-governing, benefit from volunteer activities); primary care; public hospital outreach; public community centre; home; multiple (specify); and other (specify). The profession(s) of staff providing the intervention (including volunteers, if used) and the number and characteristics (gender, age, health condition) of the recipients of the intervention were extracted.

To answer our research questions, we extracted the following data.

#### Q1. How is readiness defined and measured?

We extracted the definition of readiness (along with a key citation for the definition) and a description of how readiness was measured. For measurement of readiness we included: instrument type (readiness-specific instrument, general implementation instrument that includes a readiness component, qualitative data coded to a readiness framework); instrument name; key citation for the instrument; if the instrument is based on any other tool(s) that measure readiness; implementation science theory, model, or framework related to the instrument; a list of, and total number of, subscales and items included in the instrument; and any measures of the instrument’s psychometric properties. We used the taxonomy of Nilsen and colleagues to categorise implementation science theories, models, and frameworks (i.e., process models, determinant frameworks, classic theories, implementation theories, evaluation frameworks) [36]. If qualitative methods were used to assess readiness, we extracted the readiness framework that informed the design of the interview guide or key readiness constructs that were assessed during interviews or coded in the analyses of qualitative data (see Appendix 3 in the supplementary file [30]).

We mapped item concepts using the categories defined by Holt et al [37]. We evaluated the psychometric and pragmatic properties of each measurement instrument using the 14-criteria Psychometric and Pragmatic Evidence Rating Scale (PAPERS) [38]. The nine psychometric properties are: reliability or internal consistency; convergent construct validity; discriminant construct validity; known groups construct validity; predictive criterion validity; concurrent criterion validity; dimensionality or structural validity; responsiveness; and norms. The five pragmatic properties are: cost; language; ease of training; easy to interpret; and length. Each criterion is rated on a 6-point scale ranging from −1 (‘poor’) to 4 (‘excellent’), with 0 representing ‘not assessed’. Where possible, a single reviewer downloaded ratings from systematic reviews that evaluated measurement instruments for readiness for change that used PAPERS [1, 39]. If PAPERS ratings were not available for an instrument, a single reviewer generated ratings for the instrument.

#### Q2. What strategies are used to build readiness?

We extracted strategies used to build readiness (free text from articles). As per our subquestions, we also extracted data that referred to: timepoint(s) in the implementation process when strategies were implemented and how strategies were selected, tailored, and implemented. We also recorded whether implementation support was provided by an internal or external organisation and (if external) the type of organisation that provided support (protocol amendment).

We used three systems to categorise strategies [15]. First, we used 55 strategies for addressing organisational change [2] from the ERIC taxonomy [15]. Second, we used nine categories identified by hierarchical cluster analysis of the ERIC taxonomy [40]. This dual approach was described in a scoping review of strategies used for digital rehabilitation interventions [41]. We adapted the term ‘stakeholder’ to ‘interest-holder’ based on its colonial connotations (e.g., *develop interest-holder interrelationships*, *train and educate interest-holders*) [42]. Third, we coded each discrete strategy to one of the three domains (i.e., motivation, general capacity, innovation-specific capacity) referred to in the R = MC^2^ heuristic [11]. 

#### Q3. What is the relationship between strategies and outcomes?

We extracted data that described the relationship between strategies and outcomes (a. implementation and service-level; b. person-level). We classified implementation outcomes using a proposed minimum data set of indicators (i.e., adoption, dose delivered, reach, fidelity (adherence), sustainability (maintenance)) [21]. Service- and person-level outcomes were classified as per Proctor et al [43]. Service-level outcomes were efficiency, safety, effectiveness, equity, patient-centredness, and timeliness. Person-level outcomes were satisfaction (participation, quality of life), function (activity), and symptomatology (impairment). We extracted a description of the result for each relevant outcome for each study; the extraction included data and statistical comparisons. Results were categorised as *favourable*, *nonsignificant*, or *unfavourable* using a classification system utili*s*ed in previous scoping reviews [44, 45]. *Favourable* was used when the relationship between strategies and outcomes was positive and/or reached statistical significance. *Nonsignificant* was used when there was no relationship between strategies and outcomes and/or where the extracted results that did not reach statistical significance. *Unfavourable* was used when there was a negative relationship between strategies and outcomes and, if reported, the extracted result reached statistical significance.

### Data synthesis and presentation

The flow of references through the review is summarised in a PRISMA flow diagram generated using Covidence. We calculated frequencies for variables related to the study design, public health intervention, setting, providers, and recipients. For Q1, we tabulated definitions of readiness, characteristics of the measurement instruments, and plotted the psychometric and pragmatic properties. For Q2, we tabulated strategies as per the nine categories identified by hierarchical cluster analysis of the ERIC taxonomy [40] and calculated frequencies for how the strategies were selected, tailored, and enacted. For Q3, we counted the number of *favourable*, *nonsignificant*, and *unfavourable* results for implementation, service-level, and person-level outcomes for the packages of strategies delivered in the studies. We also tallied the relationship between categories of strategies (identified by hierarchical cluster analysis of the ERIC taxonomy [40] and ‘other’) delivered in the studies and the results (*favourable*, *nonsignificant*, *unfavourable*) for the reported outcomes. If a study used strategies from more than one category, we counted the outcome(s) for each category.

## Results

### Flow of references through the review

**Fig. 1:**
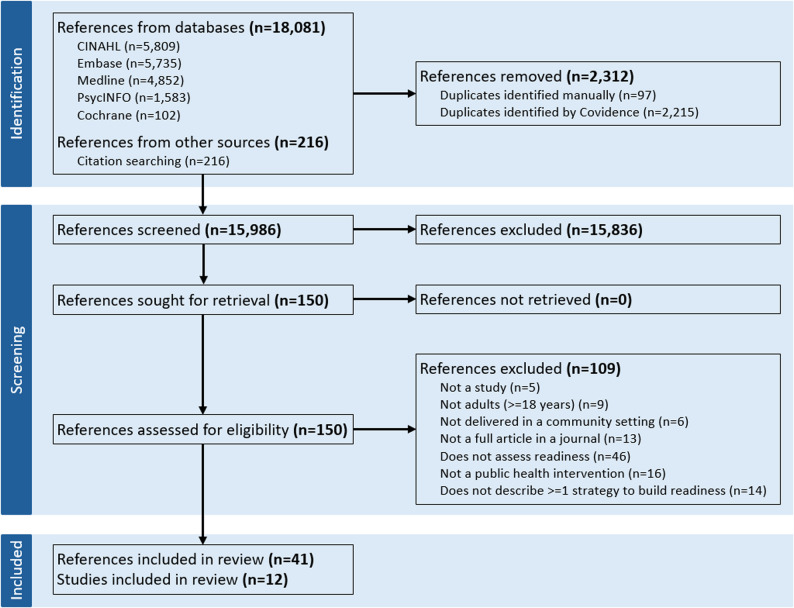
PRISMA diagram of flow of references through the review

Our search retrieved 18,297 references (Fig. 1). Of these, we removed 2,312 duplicates and excluded 15,836 in the title and abstract screening phase. Another 109 were excluded in the full text screening phase, with the main reason for exclusion being not assessing readiness (*n* = 46; reason for exclusion for each reference are in Appendix 4 in the supplementary file [30]). Forty-one references representing 12 studies met our eligibility criteria and were included in the review (all references in Appendix 5 in the supplementary file [30]) [46–57]. 

### Characteristics of the studies included in the review

All studies were published in English and were conducted in three countries (USA [46–48–49,57,52−] *n* = 10; Australia [51] *n* = 1; Spain [50] *n* = 1). Four research designs were used (group pre- versus post-test (*n* = 5) [49, 51, 54, 56, 57], randomised controlled trial (*n* = 3) [48, 52, 53], cross-sectional (*n* = 3) [47, 50, 55], non-randomised controlled trial (*n* = 1) [46]). Three-quarters of the studies had an embedded qualitative component [46–48, 50, 53–57]. 

The types of public health EBIs were physical activity (*n* = 3) [49, 54, 55], smoking cessation (*n* = 2) [51, 52], combined behavioural nutrition, physical activity and smoking cessation (*n* = 2) [47, 50], diabetes prevention (*n* = 2) [46, 56], falls prevention and chronic disease self-management (*n* = 1) [48], preventing admission to institutional care (*n* = 1) [53], and human immunodeficiency virus (HIV) prevention (*n* = 1) [57]. Interventions were implemented across different settings for seven studies (included third sector, public hospital outreach, public community centre, private sector) [48, 51–55, 57]. Three studies were conducted in primary care [46, 47, 50], one in faith- and community-based groups [49], and one in a public community centre [56]. EBIs were implemented in 4 sites [50] to 18 sites [53] in 11 studies; one study implemented the intervention in 243 sites [51]. A description of who delivered the intervention was not specified in three studies [47–49], and was delivered by case workers (*n* = 2) [51, 57], mix of different health professionals (*n* = 3) [50, 52, 53], health and wellness promotion staff (*n* = 1) [56], fitness instructors (*n* = 1) [54], nutritionists (*n* = 1) [46], and volunteers (*n* = 1) [55] in nine studies.

Nearly 40,000 people received the public health EBIs (*n* = 37,883). Nine studies included 88 participants [56] to 622 participants [51]; three studies included 5,321 [47], 7,033 [53], and 22,459 [50] participants, respectively. Most participants were women (54%; not reported in three studies [47, 51, 55]). Seven studies recruited adults (18–64 years) and older adults (≥ 65 years) [48, 53–56], four recruited adults (*n* = 4) [49, 51, 52, 57], and one recruited adolescents (< 18 years) and adults [50]. The participants were people with or trying to avoid chronic health conditions (*n* = 4 studies) [48, 54, 56, 57], institutionalisation (*n* = 1) [53] or who sought to increase their physical activity (*n* = 2) [49, 55]. Five studies focused on people seeking primary health care [50], seeking mental health services [51, 52], or military veterans [46, 47]. 

### Q1. How is readiness defined and measured?

Eight studies did not define readiness [46, 47, 49–51, 54, 56, 57], and four studies used different definitions of readiness (see Table 2). These definitions included both the structural aspects of an organisation and whether individuals within an organisation have the capacity and willingness to implement an EBI.

**Table 2:** Definitions of readiness in studies that evaluated the impact of strategies to build readiness in community-based organisations that implemented public health evidence-based interventions for adults and older adults. The primary source of each definition is in rounded brackets

“organizational climate (i.e., beliefs of the organization established by organization leaders) … organizational culture (i.e., assumptions, values, and behavioral norms) … and … organizational capacity (i.e., the adequacy of resources to meet organizational goals)” [52, (58, 59)] “the extent to which staff within an organization are psychologically and behaviorally prepared to implement a change in their setting” [53, (60, 61)] “a comprehensive attitude influenced simultaneously by the nature of change, the change process, the organization’s context, and the attributes of individuals” [17, (48)] define capacity building as “(1) rendering an organization more able to address current and future health issues, (2) creating increased problem-solving capabilities (e.g., enhancing an organization’s community engagement network or health promotion expertise), or (3) improving organizational skills, motivations, knowledge, or attitudes toward implementing innovations” [55, (62–64)]	

**Fig. 2:**
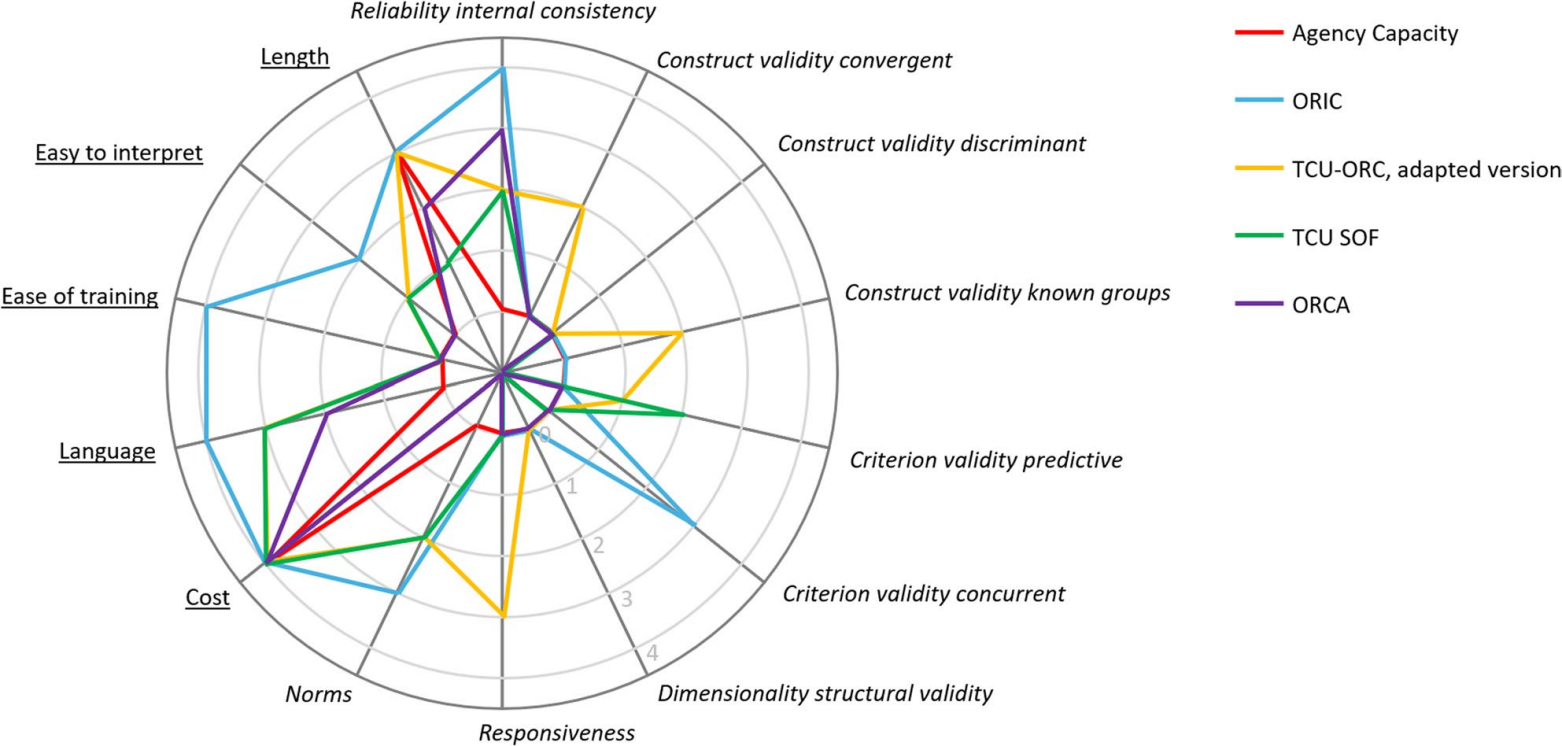
Psychometric and pragmatic properties of readiness-specific instruments used to measure readiness in the included studies. We rated psychometric properties (italicised) and pragmatic properties (underlined) using the Psychometric and Pragmatic Evidence Rating Scale (PAPERS) tool. PAPERS has a value range of −1 (‘poor’) to 4 (‘excellent’), with 0 representing ‘not assessed’. The midpoint of the figure represents a rating of −1 on the PAPERS tool. Instrument name abbreviations are: Organizational Readiness for Implementing Change (ORIC); Texas Christian University Organizational Readiness for Change Scale (TCU-ORC); Texas Christian University Survey of Organizational Functioning (TCU SOF); and Organizational Readiness to Change Assessment (ORCA)

Six studies used qualitative interviews to assess readiness [48, 50, 54–56], with all but one study using a readiness framework to inform the design or analysis [48] (see Appendix 3 in the supplementary file [30]). One study used a general implementation instrument (i.e., two questions on a staff attitudes questionnaire) [51]. Five studies used five different readiness-specific instruments, see Table 3. For example, LaBreche et al. [49] used the Agency Capacity instrument to evaluate readiness for a community- or faith-based physical activity program and Schnoll et al. [52] used the Organizational Readiness for Implementing Change (ORIC) instrument in community mental health clinics providing a smoking cessation program. The characteristics of the five readiness-specific instruments are summarised in Table 3 and the psychometric and pragmatic properties (i.e., PAPERS [38] ratings) for each instrument are illustrated in Fig. 2.

**Table 3:** Readiness-specific instruments used to measure readiness in the included studies

Measurement instrument	Model, theory or framework^*^	*N* subscales	*N* items	Item constructs^ǂ^ (%)				PAPERS total score (/56)
				Organisational structural	Organisational psychological	Individual structural	Individual psychological	
Agency Capacity [49]	Not reported	7	26	100%	0%	0%	0%	7
Organizational Readiness for Implementing Change (ORIC) [65]	Implementation theory	2	12	8%	17%	33%	42%	27
Texas Christian University Organizational Readiness for Change Scale (TCU-ORC) [66], adapted version	Classic theory	5	23^¶^	61%	9%	21%	9%	23
Texas Christian University Survey of Organizational Functioning (TCU SOF) [67]	Classic theory	31	162	46%	15%	23%	16%	13
Organizational Readiness to Change Assessment (ORCA) [68]	Determinant framework	19	74	80%	9%	4%	7%	9

^*^ Model, theory or framework was categorised according to Nilsen [36]

^ǂ^ Item constructs were categorised according to Holt et al. [37]

^¶^ full TCU-ORC has 124 items

In terms of characteristics (Table 3), two instruments measuring readiness were based on a classic theory [66, 67], one on an implementation theory [65], one on a determinant framework [68], and the theory or framework could not be determined for the Agency Capacity instrument [49]. Instruments had between 12 items [65] and 162 items [67], clustered into 2 subscales [65] to 31 subscales [67]. With the exception of ORIC [65], mapping item concepts [37] revealed a heavy emphasis on organisational structural concepts (i.e., circumstances within the organisation like policies or resources). The percentage of items mapped as *organisational structural* ranged from 46% for the TCU SOF [67] to 100% for Agency Capacity [49].

The PAPERS total score ranged from 7 points [49] to 27 points [65, 66] out of 56 (see Table 3), with higher scores indicating more substantial pragmatic and psychometric properties. In general, ratings were higher for pragmatic properties than for psychometric properties (see Fig. 2). Among pragmatic properties, cost was ranked highest (scoring 4 out of 4 for all instruments because they were free and publicly available), followed by language and length (2.4 each), and ease of training and easy to interpret (0.8 each). For psychometric properties, reliability or internal consistency had the highest mean score (2.2 out of 4), followed by norms (1.2), predictive and concurrent criterion validity and responsiveness (0.6 each), convergent construct validity (0.5), discriminant and known groups construct validity (0.0 each), and dimensionality or structural validity (−0.2).

### Q2. What strategies are used to build readiness?

The studies utilised 117 individual strategies to build readiness, ranging from 4 strategies [56] to 20 strategies [48] per study. We detail the individual strategies used in each study in the data file [30]. Using R = MC^2^ [, 11] 10 strategies focused on building *motivation to implement an innovation* (9%), 19 on increasing *general capacities of an organisation* (16%), and 88 on building *innovation-specific capacities needed for a particular innovation* (75%).

All nine categories identified by hierarchical cluster analysis of the ERIC taxonomy [40] were used in at least one study (see Table 4). We were unable to classify one strategy using the ERIC taxonomy (i.e., ‘organisational stability’), which was coded as ‘other’ [48]. Most commonly used strategies fell under the categories: *develop interest-holder interrelationships* (used 8 of 15 ERIC strategies in this category; *n* = 12 studies); *use evaluative and iterative strategies* (4 of 6 ERIC strategies; *n* = 12 studies); and *train and educate interest-holders* (5 of 8 ERIC strategies; *n* = 10 studies). For example, we extracted and coded two strategies into the *train and educate interest-holders* category from the Damschroder et al. study [46]. One was running orientation sessions for implementation leaders (coded as *conduct educational meetings* in the ERIC taxonomy [15]) and the other was providing a wide array of resources (coded as *distribute educational materials*).

**Table 4:** Number of strategy categories and number of strategies within each category used to build readiness

Study	Develop interest-holder interrelationships (8/15)	Use evaluative and iterative strategies (4/6)	Train and educate interest-holders (5/8)	Provide interactive assistance (1/1)	Change infrastructure (3/7)	Utilise financial strategies (2/7)	Adapt and tailor to context (2/4)	Engage consumers (2/4)	Support clinicians (1/3)	Other (1/1)	Strategy categories used by study (*n*)
Ford et al., 2017 [48]	7	2	3	0	1	1	1	2	1	1	**9**
Damschroder et al., 2015 [46]	4	1	2	1	1	1	2	0	0	0	**7**
Schnoll et al., 2023 [52]	2	3	3	1	1	0	0	1	0	0	**6**
Damschroder et al., 2017 [47]	6	2	2	0	1	1	0	0	0	0	**5**
Martinez et al., 2017 [50]	4	1	0	1	2	0	1	0	0	0	**5**
Vilen et al., 2022 [55]	1	1	2	1	0	1	0	0	0	0	**5**
LaBreche et al., 2016 [49]	3	1	0	1	1	0	0	0	0	0	**4**
O’Brien et al., 2012 [51]	1	1	1	0	0	1	0	0	0	0	**4**
Tomioka et al., 2013 [54]	2	3	3	0	0	0	1	0	0	0	**4**
Wilson et al., 2024 [56]	1	1	1	1	0	0	0	0	0	0	**4**
Wyatt et al., 2020 [57]	4	1	2	1	0	0	0	0	0	0	**4**
Spoelstra et al., 2022 [53]	5	4	3	0	0	0	0	0	0	0	**3**
**Studies using strategy category (n)**	**12**	**12**	**10**	**7**	**6**	**5**	**4**	**2**	**1**	**1**	

The strategy categories are the nine categories identified by hierarchical cluster analysis of the ERIC taxonomy [40] and ‘other’, with the number of ERIC strategies [15] that load into the category indicated in the heading row – the denominator is the number of ERIC strategies in each category and the numerator is the number of ERIC strategies in the category that were used in the included studies. The number of ERIC strategies are listed for each category for each study

Below we describe the most commonly used strategies within each of the three above categories.

- *Develop interest-holder interrelationships*: *develop academic partnerships* (*n* = 10 studies), *identify and prepare champions* (*n* = 8), *build a coalition* (*n* = 6), and *use advisory boards and workgroups* (*n* = 6) (see Table 2a in Appendix 6 in the supplementary file [30]).
- *Use evaluative and iterative strategies: assess for readiness and identify barriers and facilitators* (*n* = 12 studies) (see Table 2b in Appendix 6 in the supplementary file [30]).
- *Train and educate interest-holders: conduct educational meetings* (*n* = 10 studies) and *distribute educational materials* (*n* = 5) (see Table 2c in Appendix 6 in the supplementary file [30]).

Eleven studies utilised strategies in the pre-implementation and implementation phases. One study utilised strategies during pre-implementation only [51]. 

Five studies used theories, models, or frameworks to guide the selection of strategies (Social-Cognitive Theory [50], Organisational Development Theory [52], Knowledge-to-Action Model [53], Texas Christian University Program Change Model [57], theoretical program change model for translating research into practice [46]). Four studies employed strategies used successfully in other contexts (Network for the Improvement of Addiction Treatment (NIATx) [48], Diabetes Prevention Program implemented in the Veterans Health Administration [56]), or used agency guidelines (National Cancer Institute program adaptation guidelines [49], protocol developed by the Hawaii Healthy Aging Partnership [54]). Five studies tailored strategies after assessing local needs or readiness [46, 52–54, 57]. In four studies, the tailoring process was conducted through discussion with external facilitators or local change leaders [48, 50, 55, 56]. Three studies did not describe how strategies were selected [47, 51, 55] and three studies did not describe how strategies were tailored [47, 49, 51]. 

External agencies initiated and guided all studies. Five studies were led by researchers [49, 50, 53, 56, 57], three by researchers and a government agency [48, 52, 54], three by a government agency only [46, 47, 51], and one by a government agency in partnership with a consumer organisation [55]. This leadership involved selecting the participating organisations, selecting the strategies, and implementing the strategies (e.g., facilitating the networking between organisations, identifying and training local champions).

### Q3. What is the relationship between strategies and outcomes?

#### a. Implementation and service-level outcomes?

For the 12 included studies, 48 implementation or service-level outcomes were reported. Reach was the most commonly reported implementation outcome (*n* = 8 studies; *n* = 11 outcomes), followed by sustainability (*n* = 5 studies; *n* = 6 outcomes), adoption (*n* = 4 studies; *n* = 4 outcomes), fidelity (*n* = 4 studies; *n* = 5 outcomes), and dose (*n* = 3; *n* = 4 outcomes). Service-level outcomes were reported less often, with efficiency being most reported (*n* = 5 studies; *n* = 10 outcomes), followed by equity (*n* = 4 studies; *n* = 5 outcomes) and safety (*n* = 2 studies; *n* = 3 outcomes). No studies reported effectiveness, patient-centredness, or timeliness outcomes. Results for the 48 implementation or service-level outcomes were categorised: 38 (79%) *favourable*; 6 (13%) *nonsignificant*; and 4 (8%) *unfavourable*. We provide a full list of implementation and service-level outcomes, results and categorisations for each study in the data file [30]. 

**Fig. 3:**
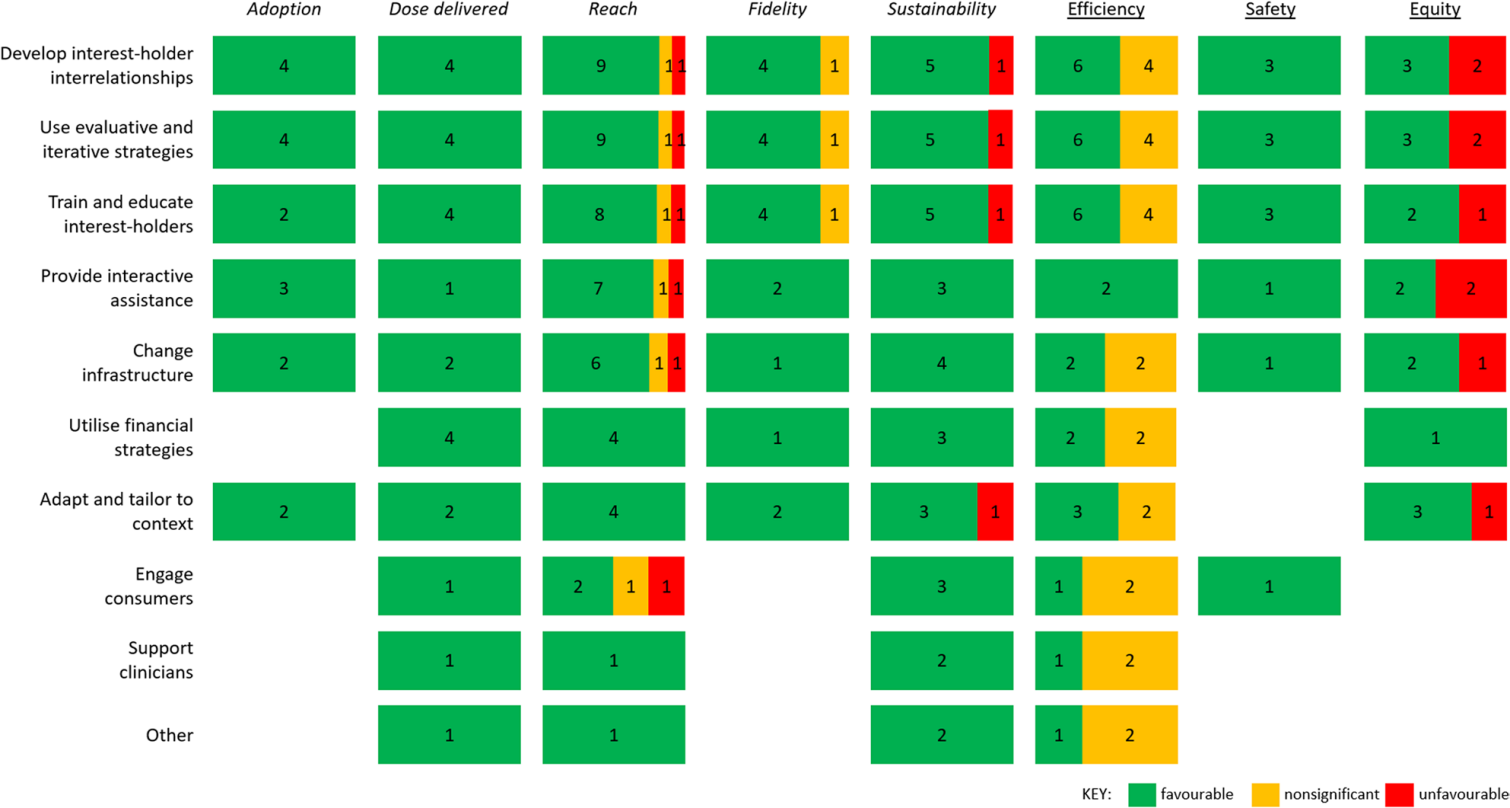
Results for implementation and service-level outcomes for each category of strategies used to build readiness. The nine categories identified by hierarchical cluster analysis of the ERIC taxonomy [40] and the ‘other’ category are listed in rows. If a study used more than one category of strategy, we counted the outcome(s) for each category. The implementation (italicised) and service-level (underlined) outcomes are in the columns. We present number of results classified as *favourable* (green), *nonsignificant* (orange), and *unfavourable* (red) for each outcome. We do not include effectiveness, patient-centredness and timeliness as these outcomes were not reported in any study

In Fig. 3 we illustrate the pattern of *favourable*, *nonsignificant*, and *unfavourable* implementation and service-level outcomes for each of the nine categories of strategies identified by hierarchical cluster analysis of the ERIC taxonomy [40] and ‘other’. If a study used strategies from more than one category of strategy, we counted the outcome(s) for each category. The dominance of green in this figure indicates that outcomes were generally *favourable*. For example, all four results for studies using the *develop interest-holder interrelationships* category of strategies [49, 50, 54, 56] were *favourable* for the adoption outcome (e.g., the proportion of organisations delivering all elements of an evidence-based physical activity program increased from 0% at baseline to 87.5% after 2 months of intervention [49]). Overall, results were all *favourable* for adoption (*n* = 17), dose delivered (*n* = 24), and safety (*n* = 12). Results were a mix of *favourable* and *nonsignificant* for fidelity (*n* = 18 *favourable*; *n* = 3 *nonsignificant*) and efficiency (*n* = 30 *favourable*; *n* = 24 *nonsignificant*); a mix of *favourable*, *nonsignificant*, and *unfavourable* for reach (*n* = 51 *favourable*; *n* = 6 *nonsignificant*; *n* = 6 *unfavourable*); and a mix of *favourable* and *unfavourable* for sustainability (*n* = 35 *favourable*, *n* = 4 *unfavourable*) and equity (*n* = 16 *favourable*, *n* = 9 *unfavourable)*. 

#### b. Person-level outcomes?

Thirty person-level outcomes were reported across 10 studies; 17 (57%) were categorised as *favourable* and 13 (43%) as *nonsignificant*. We provide person-level outcomes for each study in the data file [30]. Function was most commonly reported (*n* = 9 studies; *n* = 19 outcomes), followed by symptomatology (*n* = 6 studies; *n* = 10 outcomes) and satisfaction (*n* = 1 study; *n* = 1 outcome).

**Fig. 4:**
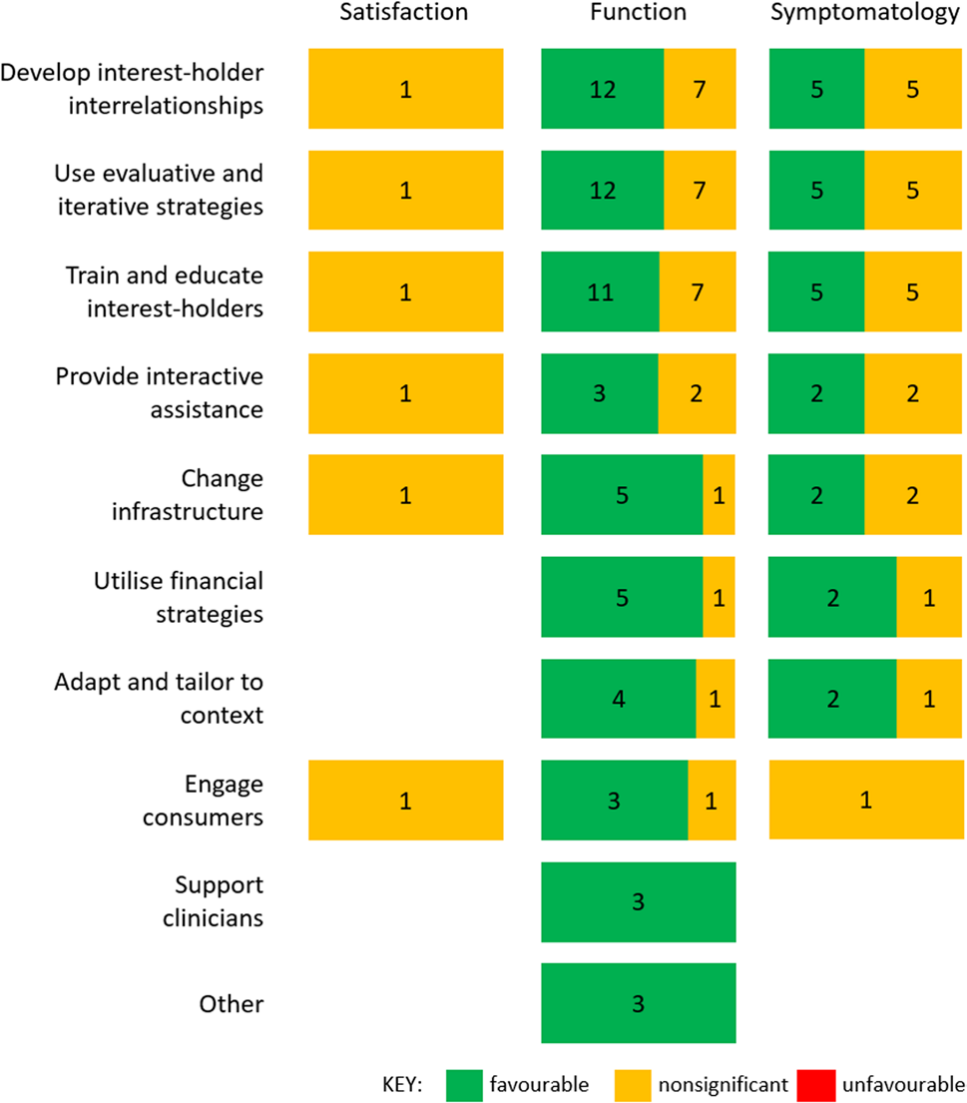
Results for person-level outcomes for each category of strategies used to build readiness. The nine categories identified by hierarchical cluster analysis of the ERIC taxonomy [40] and the ‘other’ category are listed in rows. If a study used more than one category of strategy, we counted the outcome(s) for each category. The person-level outcomes are in the columns. We present number of results classified as *favourable* (green), *nonsignificant* (orange), and *unfavourable* (red) for each outcome

In Fig. 4 we illustrate the pattern of *favourable*, *nonsignificant*, and *unfavourable* person-level outcomes for each of the nine categories of strategies identified by hierarchical cluster analysis of the ERIC taxonomy [40] and ‘other’. If a study used more than one category of strategy, we counted the outcome(s) for each category. In contrast to the implementation and service-level outcomes, the equal dominance of green and orange in Fig. 4 indicates a mix of *favourable* and *nonsignificant* results. For example, for studies that used the *engage consumers* category of strategies [48, 52], three results were *favourable* for the function outcome (e.g., the number of falls was lower in the 6 months after participating in Stepping On workshops compared to the 6 months before participation with a mean reduction of −0.429 falls/participant (*p* < 0.001 [48])) and one result was *nonsignificant* (i.e., there were no group-by-time interactions for smoking rate (*p* = 0.66) [52]). Overall, results were a mix of *favourable* and *nonsignificant* for function (*n* = 61 *favourable*; *n* = 27 *nonsignificant*) and symptomatology (*n* = 23 *favourable*; *n* = 22 *nonsignificant*). Results were all *nonsignificant* for satisfaction (*n* = 6). 

## Discussion

Our scoping review of 12 studies (mostly conducted in the USA) is the first to investigate the nature and impact of readiness-building strategies on implementing a mix of public health EBIs in various community settings. It is of concern that only one-third of studies defined readiness; of these, all studies used different definitions. There was no consensus on how readiness was measured. Only five studies used readiness-specific instruments, which all differed, and mostly focused on organisational structure and had poor psychometric properties. All studies used multiple strategies delivered by external support teams. All but one of the individual strategies used could be categorised using the ERIC taxonomy. Only two studies used the ERIC taxonomy to describe their strategies and less than half of studies described a theory, model or framework that guided strategy selection and tailored strategies to suit local context. Exploring the impact of strategies on outcomes indicated more *favourable* compared with *unfavourable* results, particularly for implementation and service-level outcomes (79% *favourable*). However, this needs to be interpreted with caution as results cannot be attributed to specific strategies or if a certain level of readiness is required for successful implementation.

Advancing ‘readiness-building’ is currently hampered by terminology that is seldom well-defined or robustly measured. As found in our review, and consistent with other literature, readiness is poorly defined in implementation science [8]. Conducting a consensus study utilising the Delphi process [69] might help to establish a global definition of readiness. A common language would support meaningful dialogue, promote replication studies and simplify data interpretation.

It is difficult to develop tools to measure readiness if definitions of what comprises effective readiness-building are inconsistent. Of the instruments used to assess readiness in our review, the ORIC tool [65] represented the broadest distribution of item constructs (8% organisational structural, 17% organisational psychological, 33% individual structural, 42% individual psychological) and had the most robust measurement properties (PAPERS total score 27 out of 56). However, as psychometric properties like validity and responsiveness have not been tested in most instruments, it is challenging to assess the effectiveness of strategies to build readiness. Conducting a consensus study [69] to select appropriate tools that measure constructs included in a standardised definition of readiness and evaluating the pragmatic and psychometric properties of readiness instruments would move the field forward. These instruments are essential to more accurately determine whether building readiness improves implementation of public health (and other) EBIs.

The studies included in our review used 117 strategies to build readiness. However, strategy use was highly variable, often not supported by theory, and most often targeted at innovation-specific capacity. The most frequently cited categories of strategies were *develop interest-holder interrelationships*, *use evaluative and iterative strategies*, and *train and educate interest-holders*. This is not surprising as these strategies are considered foundational elements of capacity building and essential to implementation success [40]. All but one strategy (‘organisational stability’ [48]) could be categorised using the ERIC taxonomy. Workforce turnover of those providing EBIs generated an increased need for training, loss of organisational knowledge, lack of intervention fidelity, and financial stress [70]. Given the challenge and implications of workforce turnover in the health system [71], assessing organisational instability as part of an organisation’s general capacity and selecting strategies that prepare for workforce turnover are likely essential readiness activities.

Fewer than half of the studies we reviewed described a theory, model or framework that guided the selection of readiness-building strategies. A similar absence of theories, models and frameworks designed to guide implementation and scale-up was noted in a review of physical activity interventions for older adults [72]. Also, although considered best practice for implementation and scale-up [73], strategies were seldom tailored to the local context [74]. Of the 12 studies we reviewed, five tailored their strategies after assessing local needs or readiness and four tailored strategies based on discussions with external facilitators or local change leaders. Strategies that target the most important determinants for practice change in a particular setting are most effective [74]. However, tailoring can be challenging from a resource perspective, particularly when scaling up to multiple organisations. Clear guidance on how best to tailor for diverse groups is currently absent from the literature [74]. 

As all studies reported using packages of strategies, it was not possible to discern the impact of any one strategy. This has implications for planning, tailoring, and optimising readiness-building strategies. Until specific drivers of readiness-building (and implementation success) are identified, a shotgun approach to choosing strategies remains a (costly) alternative. Importantly, it was also not possible to discern if a certain level of readiness is required for successful implementation. That is, what constitutes an effective, parsimonious readiness-building approach is not yet clear—but likely varies across organisations. To tease out the role of specific strategies to build readiness and promote positive person-level benefits, newer more agile trial designs are needed such as sequential multiple assignment randomised trials [75], using prospective implementation mapping of strategies to outcomes [76] with pre-planned mechanistic analyses [77]. 

All readiness-building strategies identified in our review were facilitated by external support teams. Although studies did not provide reasons for this reliance, externalising readiness building suggests that organisations lacked the internal expertise, capacity, or infrastructure they needed to implement an EBI [11]. This lack of internal capacity poses challenges for sustaining public health EBIs within an organisation. It also limits the impetus of successful implementation onto other relevant EBIs for that organisation [63]. Implementation support systems bridge the gap between research knowledge and real-world practice [78–80]. Support systems identify deficits in readiness and work with organisations to tailor appropriate strategies to build readiness. Yet little is known about the roles that support systems play in enhancing readiness [78–80]. As implementation support systems are vital to readiness building [79, 81], there is an urgent need to evaluate the contributions they make to implementation success, define their role across implementation phases, and establish dedicated funding and infrastructure to support this emerging domain of implementation practice [82]. Studies that identify internal structures that need to be in place to support effective readiness building in public health would also be of value [83]. 

Finally, there remains a tension between how to equitably balance investments in organisations that are more apt to achieve ‘readiness’ (often better resourced) versus investing more time to nurture less ready organisations (often under resourced) [54]. Researchers must be sensitised to the risk of exacerbating current inequities by underrepresenting poorly resourced groups that are most often marginalised. Tailored support that enhances readiness of *all* organisations is essential to tackle health inequities [84]. Thoughtful research that explores questions of equitable implementation support is needed [84, 85]. 

The strengths of our review include utilising a priori protocols and adhering to reporting standards, with our review meeting all PRISMA-ScR criteria [27]. We also used comprehensive search methods to capture relevant articles and used rigorous processes to screen them. However, relevant studies may have been missed due to inconsistent terms used to describe readiness.

Our data extraction processes were also rigorous. However, the low-level of detail used to describe the readiness-building strategies and lack of use of standardised terminology (e.g., only two included studies used the ERIC taxonomy [15] to describe their strategies [47, 52]) made data extraction challenging. This may have limited how accurately we were able to identify and report strategies used to build readiness. We urge authors and journal editors to use the ERIC taxonomy [15] to name strategies, to provide sufficient detail to operationalise the strategies [86], and use contemporary reporting standards to guide publication (e.g., Standards for Reporting Implementation Studies (StaRI) [87]). This will more readily enable studies to be replicated and for outcomes to be translated into practice. Finally, the complexity of how best to present data on the impact of readiness-building strategies on outcomes might be considered a limitation. Although there is no straight-forward way, we chose a reporting system that has been used in previous scoping reviews [44, 45] and deemed to be easily understandable for readers. We acknowledge that there may be better ways to visually present our findings.

## Conclusions

The results of this review indicate that strategies are used to build readiness to implement public health EBIs in community-based organisations; however, most target readiness for a specific public health EBI (i.e., innovation-specific capacity versus motivation and general capacity) and are enacted by external support teams. Policymakers and funders should consider how to offer ongoing funding for support teams (internal or external to organisations) to enable sustainment of EBIs and how general organisational capacity and motivation can be built for broader impact. Key recommendations for future research are to establish consensus on the definition of readiness, develop validated and well-tested tools for measuring change in readiness, and the use of research designs and analysis that enable identification of key readiness-building strategies and their mechanism(s) of action.

## Supplementary Information

Supplementary Material 1.

## Data Availability

The datasets and supplementary materials supporting the conclusions of this article are available in a public, open access repository (Sydney eScholarship Repository, https://hdl.handle.net/2123/34540, Creative Commons Attribution 4.0 Licence) [30].

## Abbreviations

CFIR: Consolidated Framework for Implementation Research (CFIR)
EBI: Evidence-based intervention
ERIC: Expert Recommendations for Implementing Change
NAITx: Network for the Improvement of Addiction Treatment
ORCA: Organizational Readiness to Change Assessment
ORIC: Organizational Readiness for Implementing Change
PAPERS: Psychometric and Pragmatic Evidence Rating Scale
PRISMA-ScR: Preferred Reporting Items for Systematic reviews and Meta-Analyses extension for Scoping Reviews Statement
R: Readiness in R = MC^2^ heuristic
M: Organisation’s motivation in R = MC^2^ heuristic
C: General capacities in R = MC^2^ heuristic
C: Innovation-specific capacities in R = MC^2^ heuristic
STaRI: Standards for Reporting Implementation Studies
TCU SOF: Texas Christian University Survey of Organizational Functioning
TCU-ORC: Texas Christian University Organizational Readiness for Change Scale
